# Functional mapping of thalamic nuclei and their integration into cortico-striatal-thalamo-cortical loops via ultra-high resolution imaging—*from animal anatomy to in vivo imaging in humans*

**DOI:** 10.3389/fnins.2013.00024

**Published:** 2013-05-08

**Authors:** Coraline D. Metzger, Ysbrand D. van der Werf, Martin Walter

**Affiliations:** ^1^Clinical Affective Neuroimaging Laboratory, Department of Psychiatry and Psychotherapy, Center for Behavioral Brain Sciences, Otto-von-Guericke UniversityMagdeburg, Germany; ^2^Department of Behavioral Neurology, Leibniz Institute for NeurobiologyMagdeburg, Germany; ^3^Department of Sleep and Cognition, Netherlands Institute for Neuroscience, an Institute of the Royal Netherlands Academy of Arts and SciencesAmsterdam, Netherlands; ^4^Department of Anatomy and Neurosciences, VU University Medical CenterAmsterdam, Netherlands

**Keywords:** thalamus, functional brain networks, thalamo-cortical circuits, high-resolution imaging, ultra high field fMRI

## Abstract

The thalamus, a crucial node in the well-described **cortico-striatal-thalamo-cortical circuits**, has been the focus of functional and structural imaging studies investigating human emotion, cognition and memory. Invasive work in animals and post-mortem investigations have revealed the rich cytoarchitectonics and functional specificity of the thalamus. Given current restrictions in the spatial resolution of non-invasive imaging modalities, there is, however, a translational gap between functional and structural information on these circuits in humans and animals as well as between histological and cellular evidence and their relationship to psychological functioning. With the advance of higher field strengths for MR approaches, better spatial resolution is now available promising to overcome this conceptual problem. We here review these two levels, which exist for both neuroscientific and clinical investigations, and then focus on current attempts to overcome conceptual boundaries of these observations with the help of **ultra-high resolution imaging**.

## The role of the thalamus in cortico-subcortical network integration

Functional imaging studies of the last decade have thoroughly investigated the importance of cortical networks in highly differentiated subfunctions of human behavior. Interaction, co-play and functional alterations of these networks have been related to various higher order cognitive functions, behavior and disease. While a continuing debate on segregated networks generally focuses on cortical regions (Barabasi and Albert, 1999; Hagmann et al., 2008; Bullmore and Sporns, 2009), the interaction of subcortical structures—foremost thalamus and basal ganglia—with these cortical networks, their influence and control has hardly been investigated and therefore remains poorly understood. Such a cortico-subcortical interaction seems, however, likely given anatomical and stimulation studies in animals (for review see Haber, 2003) and post-mortem studies in humans (reviewed by Romanelli et al., 2005) and has been previously suggested by Alexander and colleagues (1986). Moreover, cortical-subcortical interaction have been indicated to be especially important for fear-conditioning and emotional behavior (e.g., LeDoux, 1993; Price and Drevets, 2010). Due to the invasiveness of most functional investigations of such cortico-subcortical networks, studies in humans are so far limited to methods such as functional magnetic resonance imaging (fMRI) or—in very rare cases—deep brain stimulation (DBS). For fMRI, insufficient spatial resolution in most studies limited the interpretation of thalamic activation, while continuous innovation in high resolution fMRI (hr-fMRI) now enables the functional investigation of small, anatomically well-described subcortical structures including the thalamus—also in humans. The thalamus has been described as the “gate to the cortex,” encompassing well-characterized, anatomically and functionally segregated nuclei as described in a massive amount of animal (Alexander et al., 1986; McFarland and Haber, 2002; Zikopoulos and Barbas, 2007; Xiao et al., 2009) and human post-mortem studies (Cullen et al., 2003; Danos et al., 2003; Pakkenberg et al., 2009). From these studies, it is known that thalamic nuclei are embedded in so-called cortico-striatal-thalamo-cortical circuits or “loops” (Alexander et al., 1986), making the thalamus a major relay station for cortical input.

The high specificity and behavioral relevance of these circuits in humans makes most invasive neuroanatomical approaches in the human central nervous system impossible and the study of these circuits thus highly depends on translations between species. With the aid of the new techniques of hr-fMRI, the functional investigation of such cortico-subcortical interactions and related follow-up studies are now possible. This review focuses on the description and discussion of specific thalamic imaging findings in high-resolution fMRI in humans and their embedding in brain network interaction by giving the reader a large overview on specific and unspecific thalamic findings in animals and humans.

## Previous functional findings in thalamic regions at lower fields

Previous descriptions of thalamic activation can be found in a large number of human imaging studies of cognition (Minzenberg et al., 2009; Ide and Li, 2011; Li et al., 2012), emotion (Mouras et al., 2003; Moulier et al., 2006; Kober et al., 2008; Kessler et al., 2011; Kühn and Gallinat, 2011, 2012; Sabatinelli et al., 2011; Grimm et al., 2012), health and disease (Dickstein et al., 2006; Maisog et al., 2008; Ide and Li, 2011; Passamonti et al., 2011).

### Thalamic findings related to cognition

Thalamic activations have not been consistently reported at conventional field strengths; indeed, in 2000 Cabeza and Nyberg reviewed 275 studies on positron emission tomography (PET) and fMRI data investigating cognitive processing (Cabeza and Nyberg, 2000). In processes of attention, perception, imagery, language, memory, and priming, the authors could not find consistent activation in thalamic regions among the reviewed studies. Individual studies have nevertheless reported thalamic activation, most often in conditions of attention demanding tasks, as illustrated below using various examples of functional imaging in cognitive tasks.

In one of the first studies reported, Portas and colleagues reported activation near the ventrolateral thalamus during an attentional task, which changed as a function of arousal—with highest activity under low arousal conditions (Portas et al., 1998). Increasing processing demands were moreover associated with increased thalamic activation during several tasks (Adler et al., 2001). Sturm and Willmes, based on own prior work and functional MRI studies proposed an involvement of the thalamus in a right lateralized network (including frontal, parietal, thalamic, and brainstem regions), driven by alerting and orienting attentional demands (Sturm and Willmes, 2001). LaBar and colleagues reported unspecific overlapping thalamic activation during both a spatial attention and a working memory task in the same subjects (LaBar et al., 1999). Such overlapping activation for attentional tasks within subjects, was also found by Adler and colleagues (Adler et al., 2001). In the specific case of auditory top-down attention, Li and colleagues reported unspecified thalamic activation that related to temporal cognition (Li et al., 2012). Ide and colleagues described a causal relationship between medial thalamic activation during error detection and activity in the ventrolateral prefrontal cortex during post detection slowing (Ide and Li, 2011). In 2008, Dosenbach and colleagues, based on graph analysis on restingstate fMRI data, proposed the anterior thalamus, to belong to a cingulo-opercular network, important for a stable maintenance of task set (Dosenbach et al., 2007, 2008). In 2009, Minzenberg and colleagues reviewed 41 neuroimaging studies about executive function in healthy and schizophrenic subjects, revealing that the left thalamus was involved in executive function, while left lateralized thalamic activation near the mediodorsal thalamus was reduced in schizophrenic patients during executive function performance (Minzenberg et al., 2009).

It appears that thalamic activation in earlier functional MRI studies has been inconsistently found, but that in various experimental designs requiring high levels of attention or complex problem solving, evidence for thalamic involvement exists.

### Thalamic findings related to emotional processing

A meta-analysis of 162 studies revealed the thalamus—specifically its mediodorsal and centromedian portions- to belong to a core limbic group of regions, most likely representing the autonomic component of emotional processing (Kober et al., 2008). During sexual arousal, Mouras and colleagues did, however, not find consistent thalamic activation (Mouras et al., 2003), while a correlation between circumferential penile response during visual erotic stimulation with activation in the right ventrolateral thalamus could be found (Moulier et al., 2006). This last finding is supported by recent meta-analystic analysis on male sexual arousal, showing consistent activation in the thalamus during erotic stimulation (Kühn and Gallinat, 2011; Stoléru et al., 2012). Moreover, thalamic activation has been linked to more general subjective positive emotional experiences in a second meta-analysis by Kühn and Gallinat (2012), in line with Sabatinelli et al. (2011), who performed a review based on 57 patients, and concluded on involvement of the mediodorsal thalamus in processing positive emotional scenes. However, thalamic responsiveness might not be restricted to processing of positive arousing stimuli. Kessler and colleagues found thalamic activation during dynamic emotional expression- irrespective of emotion in a study investigating still and dynamic facial expressions of sadness, happiness, disgust and fear (Kessler et al., 2011). This finding was recently corroborated by Grimm and colleagues (2012), who found an effect of valence in the right thalamus for emotional vs. neutral words, but also for negative vs. positive word encoding.

### Thalamic findings related to memory processing

Both animal and human data support a role for thalamic nuclei in memory processing (Aggleton et al., 2011). Such data stem from clinical studies in humans, often involving ischemic damage to regions of the thalamus, as verified by structural MRI, and from animal experimental studies showing the effects of surgical lesions to memory performance (van der Werf et al., 2000, 2003a; Mitchell et al., 2008). From these data it is clear that thalamic territories involved in memory are located in the anterior and medial parts of the thalamus. These parts contain the so-called anterior nuclei of the thalamus [the anteromedial nucleus (AM), anterodorsal nucleus (AD) and anteroventral nucleus (AV) nuclei, perhaps also the laterodorsal nucleus (LD)], the medial dorsal nucleus (MD) and the midline and intralaminar nuclei. The effect of anteriorly located thalamic lesions causing amnesia resembling that seen after lesions to the hippocampal formation have been well-described (van der Werf et al., 2003b). Such lesions typically cause anterograde amnesia characterized by a deficit of new learning and a recollection deficit. Lesions to the MD, midline and intralaminar nuclei produce less clear but still disabling effects on memory and cognition (van der Werf et al., 1999; Pergola et al., 2012). Consequences of such lesions have been described as following from deficits of strategic recall. The involvement of the anterior and medial parts of the thalamus agrees with their known neuroanatomical connections (van der Werf et al., 2003a): the anterior nuclei receive inputs from the mammillary bodies that in turn receive strong projections from the hippocampal formation. Their output is directed toward the different parts of the cingulate cortex. As such, they are part of the Delay-Brion circuit. The medial dorsal nucleus sends and receives strong projections to the prefrontal cortex, but also receives inputs from medial temporal lobe structures. The midline and intralaminar nuclei are small nuclei with a widespread projection pattern with the rest of the brain and inputs mainly from brain stem and subcortical areas (van der Werf et al., 2002). Specific roles of these nuclei in processes of memory have been difficult to establish due to their small size and poor resolution using conventional field strength MRI.

An influential paper by Aggleton and Brown (1999) described how different types of memory are subserved by parallel circuits involving the thalamus: a hippocampal-anterior thalamic circuit mediating recall or recollection and a perirhinal–medial dorsal thalamic circuit for familiarity judgments. This proposal has spawned a great deal of investigation, with some studies corroborating and some contradicting the idea of a separation of memory functions at the level of the thalamus (Zoppelt et al., 2003; Kishiyama et al., 2005; Carlesimo et al., 2007; Cipolotti et al., 2008). Aggleton and co-workers have recently nuanced their original proposal and stated that the role of the medial dorsal nucleus of the thalamus may lie in effects upon both recollective-based and familiarity-based recognition (Aggleton et al., 2010). Apart from the more common anterograde amnesia seen after thalamic damage, a rare and intriguing memory deficit sometimes occurs, i.e., retrograde amnesia. Lesions underlying such symptoms again often involve the medial dorsal nucleus and would result from deficits of coordinated recall, governed by the interplay between the medial dorsal nucleus and the prefrontal cortex (Miller et al., 2001). A challenge for the study of thalamic involvement of thalamic substructures in memory processes for the coming years is to attribute specific memory types or sub-processes to individual nuclei, groups of nuclei, or even subparts of nuclei, using the possibilities offered by high field strength imaging combined with sensitive memory paradigms.

### From low to high field strength

Insufficient resolution in most of the mentioned studies on cognition, emotion and memory has limited the interpretation the described thalamic findings and their attribution to specific thalamic nuclei. Therefore, this activation was often addressed to one of the larger thalamic nuclei like the mediodorsal nucleus (e.g., Walter et al., 2008a; Grimm et al., 2009) or not specified at all. The involvement of “the thalamus” as a whole in one subcomponent of human behavior—like cognition or emotion—might be oversimplified given its established functional heterogeneity in animals. Therefore, some studies have aimed to specify thalamic activation with respect to certain nuclei, mainly based on localized maximum peak activation, situated in large thalamic relays nuclei—e.g., for the mediodorsal and ventrolateral thalamic nucleus in emotional and erotic processing (Walter et al., 2008a; Abler et al., 2011)—or based on co-affected network structures e.g., for alterations of the mediodorsal nucleus (together with anterior cingulate cortex) in major depressive disorder patients (e.g., Grimm et al., 2009).

In contrast to these lower field (3Tesla) studies, high-resolution imaging at high field strength enabled to look at specific thalamic nuclei, their function, anatomy and therefore functional integration in cortico-striatal-thalamo-cortical circuits.

## Current findings on thalamic nuclei using high resolution fMRI—from animal anatomy to *in vivo* functional thalamic mapping in humans

While cortico-subcortical circuits have been hypothesized to be involved in very diverse functions like movement, attention, cognition and emotion (Alexander et al., 1986), these integrated loops may account for different facets of basic human behavior. Sexual processing comprises a suitable example paradigm to investigate such diversity, given its characteristic multidimensionality. Spanning various functional systems, brain areas, and networks, processing of sexually salient information involves cognitive, motivational, emotional, and autonomic processes, which contribute to the subjective experience of sexual arousal (Redouté et al., 2000).

**Figure 1:**
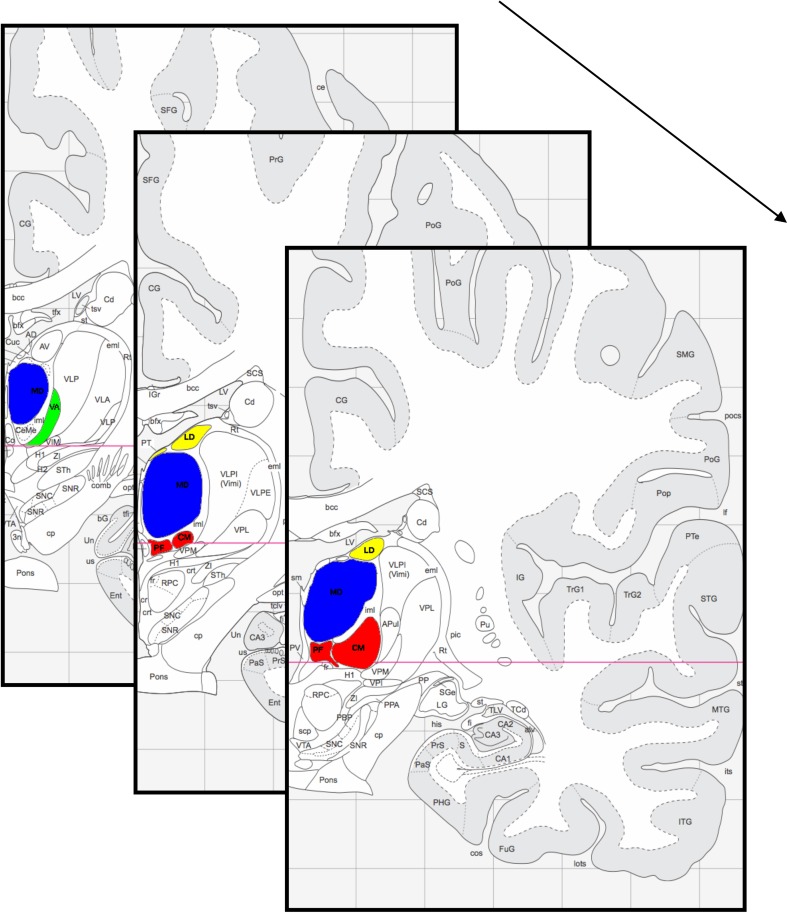
**Thalamic section from anterior (back) to posterior (front).** Highlighted are the mediodorsal thalamic nucleus (blue), the centromedian/parafascicular thalamic complex (red), the laterodorsal and parataenial thalamic nucleus (yellow) and the ventral anterior thalamic nucleus (green). Figure adapted from Mai et al. (2004).

We will therefore in the next section link findings from animal anatomical and human high-resolution studies to explain functionality and anatomy of thalamic nuclei involved in sexual processing. For (1) the mediodorsal nucleus and (2) the centromedian/parafascicular complex the animal anatomical and functional literature will be described, followed by related functional studies in humans at low field strength and consecutive studies at high-field strength. For anatomical guidance, all described nuclei are visualized in Figure 1.

### The mediodorsal thalamic nucleus processes emotionally salient information

As shown in monkeys, the **mediodorsal thalamic nucleus (MD)** is the main part of the limbic thalamus (Vogt and Pandya, 1987; Vogt et al., 1987) and forms a thalamic relay station linking basal ganglia and cortex (McFarland and Haber, 2002). The MD provides strong cortical and subcortical connections. Subcortical connections target the periaqueductal gray, ventral, and dorsal tegmental area, claustrum, the innominate substance, lateral portions of habenula and hypothalamus, caudate and amygdala as shown in monkeys (Nakano et al., 1990; Erickson and Lewis, 2004) and rats (Krettek and Price, 1977; Cornwall and Phillipson, 1988).

Cortical connections are described to the prefrontal cortex in cats, primates and also humans (Alexander and Fuster, 1973; Vogt and Pandya, 1987; Vogt et al., 1987; Price, 1999; Erickson and Lewis, 2004; Klein et al., 2010; Zhang et al., 2010; Eckert et al., 2011).

Connections of MD to the anterior insula have been described in rodents and monkeys (Reep and Winans, 1982a,b; Allen et al., 1991; Ray and Price, 1992, 1993) and so far by one study in humans (Eckert et al., 2011).

The MD was shown to play a major role in cognition, emotion, memory, and motivation in rats and monkeys [Oyoshi et al., 1996; Haber and Knutson, 2010, reviewed by Haber and McFarland (2001)].

Despite insufficient resolution, connectivity studies in humans have described the MD as part of the salience network (Seeley et al., 2007). Activation in the MD was found in studies investigating sexual processing in human males (Redouté et al., 2000; Arnow et al., 2002; Karama et al., 2002; Heinzel et al., 2006).

A recent study at low fields could differentiate regions involved in specific sexual arousal, general emotional intensity or valence (Walter et al., 2008a). While the hypothalamus and the ventral striatum form specific core structures of sexual arousal, the pregenual anterior cingulate cortex was associated with the integration of sexual intensity with emotional valence. In this context, the thalamus has been shown to mediate emotional intensity, mainly via its mediodorsal portions. Building on these findings relating to sexual processing, a study using hr-fMRI focused on the feasibility of thalamic hr-fMRI at 7 Tesla (Walter et al., 2008b). Based on the description of mediodorsal thalamic activation during emotional processing at lower fields (Walter et al., 2008a), this study investigated the individual reliability and localizability of the mediodorsal thalamic nucleus in 10 healthy individuals on single subject level at high field strength. Proving the reliable activation of the mediodorsal thalamic nucleus with the help of hr-fMRI, this was the first study, able to distinguish certain thalamic nuclei and small parts of the basal ganglia including the claustrum.

Based on this first description by Walter et al. (2008b), a follow-up study by Metzger and colleagues (2010) focused on differential effects of emotional and attentional components of sexual processing in humans on specific and unspecific thalamic nuclei, via group-level analysis at ultra high fields. While the emotional perception of sexually relevant stimuli in the mediodorsal thalamic nucleus (MD) could be confirmed, a clear distinction between these emotional processes in the mediodorsal thalamus and attentional processes (associated with the centromedian/parafascicular complex (CM/PF) of the intralaminar thalamus—as stated below) could be made.

### The intralaminar centromedian/parafascicular complex and its involvement in attentional shift and attention to salient stimuli

The intralaminar **centromedian/parafascicular thalamic complex (CM/PF)** forms the major part of the intralaminar nuclei (van der Werf et al., 2002). In animals strong connections of CM/PF—foremost of the centromedian nucleus—with the basal ganglia (Jones and Leavitt, 1974; Royce and Mourey, 1985; Cornwall and Phillipson, 1988; Berendse and Groenewegen, 1990; Nakano et al., 1990; Fenelon et al., 1991; Sadikot et al., 1992; Haber and Calzavara, 2009; Vertes et al., 2012), somatosensory, motor and premotor cortices (Berendse and Groenewegen, 1991; François et al., 1991) are described. In monkeys and rodents, the parafascicular portion of CM/PF shows strong connections to BA24 as part of the dorsal anterior cingulate cortex (dACC) (Vogt and Pandya, 1987; Vogt et al., 1987; van der Werf et al., 2002). Connections to the anterior insula have been reported in rodents (Reep and Winans, 1982a,b) and monkeys (Mufson and Mesulam, 1984) only.

Apart from its association to motor control (Smith et al., 2009) and pain (Weigel and Krauss, 2004) the CM/PF has been related to attentional processing in animal studies (Kinomura et al., 1996; Minamimoto and Kimura, 2002; Haber and Calzavara, 2009) and more specific to the attentional shift to motivationally relevant stimuli (Matsumoto et al., 2001; van der Werf et al., 2002). Being part of the ascending reticulo–thalamo–cortical activating system (Moruzzi and Magoun, 1949; Isaacson and Tanaka, 1986; Cornwall and Phillipson, 1988) and therefore mediating general arousal and the level of cortical activity (Haber and Calzavara, 2009), the CM/PF has been directly related to sexual arousal, erection and sexual behavior in rodents (Heeb and Yahr, 1996, 2001; Coolen et al., 1997, 2003; Veening and Coolen, 1998; Temel et al., 2004). Matsumoto and colleagues (2001) found CM/PF activations in monkeys to be directly related to unexpected stimuli, but not to reward.

High-resolution imaging at ultra high field strength could find activation toward such attentional processes in the intralaminar CM/PF in humans (Metzger et al., 2010), and clearly separate it from activation toward emotional stimulation (as stated above). Convergent with animal studies in which CM/PF was described to mediate the attention toward salient stimuli (Matsumoto et al., 2001), this effect could be confirmed in humans as expectancy of sexually relevant information was linked to activation in CM/PF.

It should be mentioned that in the 7T fMRI study by Metzger et al. (2010), further specific activations were reported for laterodorsal and ventral anterior thalamic nuclei. For reasons of space limitation, we would like to refer the interested reader to the original publication for further details.

## Anatomical underpinnings underlying thalamo-cortical circuits—adding *in vivo* human anatomy to the picture of thalamic function:

Thalamic connections have been extensively described in animals and are revised in the previous paragraph sorted by nuclei (Reep and Winans, 1982a,b; Mufson and Mesulam, 1984; Royce and Mourey, 1985; Vogt and Pandya, 1987; Vogt et al., 1987; Cornwall and Phillipson, 1988; Nakano et al., 1990; Berendse and Groenewegen, 1991; Sadikot et al., 1992).

A parcellation of the human thalamus based on *in vivo* imaging has developed from a rough division based on anatomical and functional connectivity (Johansen-Berg et al., 2005; Fair et al., 2010; Zhang et al., 2010) over smaller more accurate parcellations (Klein et al., 2010; Kim et al., 2012) to parcellations on single nuclei level (Eckert et al., 2011). These nuclear parcellations might have the closest link to the observations in animals.

The following paragraph will give an overview of the development of anatomical thalamic parcellation in human imaging studies over the last years.

As one of the first studies, Johansen-Berg and colleagues divided the thalamus based on fiber connections with large predefined cortical areas like the prefrontal cortex, temporal- and occipital cortex and three specific gyri like the somatosensory-, premotor- and motor cortex (Johansen-Berg et al., 2005).

A similar approach was used by Zhang and colleagues, when dividing the thalamus by its functional connectivity in resting state fMRI with the same cortical lobes and areas and comparing it to fiber counts assed by diffusion tensor imaging (DTI) in the same subjects and a human histological talairach atlas (Zhang et al., 2010). Both studies by Johansen-Berg and Zhang used very large cortical areas, therefore receiving a very rough thalamic parcellation. However, these studies added to the field in confirming anatomical knowledge from animal and human post-mortem studies by using non-invasive brain imaging in humans *in vivo* by two commonly used brain imaging techniques—namely DTI and resting state fMRI.

In line with these large-scale parcellations, Fair and colleagues specified changes of thalamic connectivity with these six cortical divisions over development and livespan (Fair et al., 2010).

Based on resting state fMRI data, Kim and colleagues parcellated the thalamus and basal ganglia in various functional subdivisions (Kim et al., 2012). Therefore, they used an independent component analysis, which groups all subregions in the brain according to their similarity of behavior during resting state. Despite using a low resolution, this study could find a functional thalamic parcellation, similarly diverse as described in human post-mortem atlases.

All three studies revealed preferential connectivity of the mediodorsal thalamic portion with the prefrontal cortex. This relationship was specified by Klein and colleagues, who divided the mediodorsal thalamus according to its **preferential connectivity** with subdivisions of the prefrontal cortex in monkeys and humans (Klein et al., 2010). They confirmed a specific diversity within the mediodorsal thalamic nucleus, which was previously reported in animal and post-mortem studies (Haber and McFarland, 2001; Haber and Calzavara, 2009).

**Figure 2:**
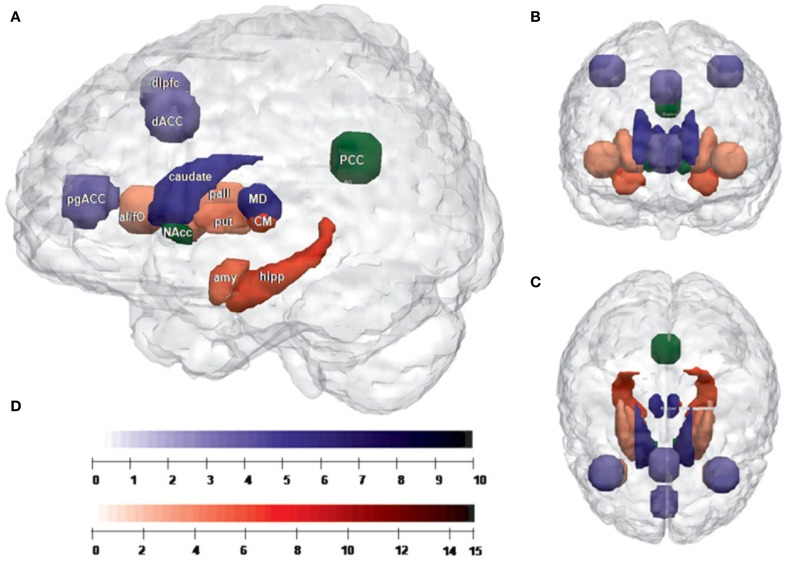
**(adapted from Eckert et al., 2011): Network segregation based on relative fiber counts. (A)** Sagittal plane; **(B)** cornal plane; **(C)** transversal plane **(D)** color bars: indicating the level of *T*-values for each region shown in **(A–C)**. Regions with preferential connectivity to the MD are shown in blue and those connecting stronger to the CM/Pf complex are shown in red, the strength of the connectivity are visualized in the brightness of the blue and red colors. The PCC and the nucleus accumbens do not show significant preferences and appear in green. Abbreviation MD, mediodorsal thalamic nucleus; CM, centromedian/parafasicualar complex of the thalamus; amy, left amygdala; hipp, left hippocampus; PCC, posterior cingulate cortex; put, right putamen; pall, right pallidum; NAcc, right nucleus accumbens; caudate, right caudate nucleus; dlPFC, right dorsolateral prefrontal cortex; dACC, dorsal anterior cingulate cortex; pgACC, pregenual anterior cingulate cortex; aI/fo, left anterior insula-frontal operculum.

Based on the findings by Metzger and colleagues, the anatomical underpinnings of the differential functional connectivity of MD and CM/PF under task circumstances were investigated by DTI (Eckert et al., 2011). Looking at preferential anatomical connectivity between MD, CM/PF and cortical as well as subcortical targets, Eckert and colleagues described a relative preference of fiber tract distributions for both nuclei in humans. While MD was preferentially connected with cortical structures (with the exception of the caudate nucleus), CM/PF showed preferential connectivity with subcortical structures, with the exception of the anterior insula (Figure 2).

Based on these preferential connectivities, this study could indicate the structural basis of putative networks, with MD connecting to prefrontal cortical areas and CM/PF interconnecting with subcortical areas. Therefore the authors suggested MD and CM/PF as two important neighboring hubs of structurally and functionally segregated networks in humans. Their result supported the segregation of thalamic nuclei into functional units of distinct cortico-subcortical systems. In this view, the involvement of the cingulate cortex and the anterior insula in distinct attentional processes like attentional set and switching of attention (Yamasaki et al., 2002; Menon and Uddin, 2010), supports the idea of cortico-striatal-thalamo-cortical loops involved in segregated but integrated circuits of information processing.

## Thalamo-cortical loops and networks

The described developments in the field of human thalamic functional connectivity *in vivo*, from unspecific findings in activation studies to more and more detailed parcellations, form the bases for ultra high resolution studies, which describe specific thalamic activation and therefore function, together with specific connectivity of certain nuclei and their integration into cortico-striatal-thalamo-cortical loops. The following paragraph gives an overview of findings in the animal literature on thalamocortical loops, and related human studies *in vivo* at low and high field strength.

The concept of segregated but integrated circuits, forming functional subsystems or cortico-striatal-thalamo-cortical “loops,” was suggested by Alexander and others, mainly based on animal studies (1986).

These cortico-striatal-thalamo-cortical loops connect functionally segregated, higher order cortical regions with the basal ganglia and form functional entities for motor and sensory processes as well as for higher cognitive functions like cognitive and emotional processes. This concept has been further put forward by research in animals, healthy humans, and diseased subjects (Price, 1999; Haber and Calzavara, 2009).

In humans such a distinction of segregated networks has been described on cortical level between two major networks, namely the default mode- and the attention network, comprising several subnetworks (Buckner et al., 2008; Biswal et al., 2010).

The attention network—or task-positive network—is always active, when a cognitive or attentionally demanding task is performed. In contrary, the default-mode network—or task-negative network is related to self-referential, interoceptive processing and is more active when no specific task is required and has been associated with increased mind-wandering. Both networks are anticorrelated, meaning high activity in one network is related to low activity in the other and vice versa.

Characteristic hubs of the task-positive network lie in the inferior parietal sulcus, superior parietal lobule and frontal eye-field for top-down control, in the temporo-parietal junction and ventral frontal cortex for stimulus-driven attention and in the anterior insula and the dorsal anterior cingulate cortex for continuous attention and salience processing (Corbetta and Shulman, 2002; Seeley et al., 2007; Dosenbach et al., 2008), while the task-negative default mode network encompasses parts of the medial prefrontal cortex (BA10), the posterior cingulate cortex extending toward the precuneus as well as temporo-parietal and lateral temporal regions.

While in humans such networks have been extensively described on cortical level, not so much is known about subcortical hubs orchestrating these cortical networks.

As there is striking evidence from a large number of animal studies that anatomical thalamic parcellation into thalamic nuclei also mirrors functional diversity (Groenewegen and Berendse, 1994; Haber, 2003), such functional integration of thalamic nuclei into cortico-striatal-thalamo-cortical loops has been proposed.

The study by Klein and colleagues focused on connectivity between anatomically predescribed subparts of the mediodorsal thalamic nucleus and subregions of the prefrontal cortex using diffusion tensor tractography (Klein et al., 2010). Using DTI in macaque and humans, they could confirm a topographical division within the MD thalamus for preferential prefrontal connectivity previously described by tracer studies in monkeys (Ray and Price, 1993). This study, however, focused on anatomical connectivity patterns. To assess functional network segregation on thalamic and basal ganglia level, more functional studies, involving both default mode- and attention network, looking at predefined thalamic nuclei would be essential. Therefore, the study by Metzger and colleagues focused on functional connectivity during task performance of specific thalamic nuclei like MD, CM/PF, VA and LD/PT, addressing the question of functional network affiliation of these nuclei (Metzger et al., 2010). They found a coactivation of CM/PF with the task-positive salience network, while MD coactivated with more rostral parts of the anterior cingulate cortex, namely the pregenual anterior cingulate cortex (pgACC), which was previously characterized to show default-mode like behavior in functional studies (Northoff et al., 2007; Grimm et al., 2009; Walter et al., 2009; Biswal et al., 2010). Moreover, and in line with the described findings by Fair and colleagues (2010), the VA showed a coactivation with the premotor cortex, also supported by anatomical VA connectivity patterns in animals (Katayama et al., 1986; Fang et al., 2006). This finding of cortico-striatal-thalamo-cortical integration was further substantiated by the anatomical findings of preferential connectivity of MD and CM/PF with the predefined network hubs like dACC, pgACC and insula (Eckert et al., 2011).

Functional connectivity, substantiated by anatomical connectivity of these cortical network hubs together with distinct thalamic nuclei and basal ganglia components thus support the distinction of affective and cognitive cortical subdivisions based on segregated cortico-striatal-thalamo-cortical loops (Devinsky et al., 1995; Bush et al., 2000).

While these studies form a first step toward the discovery of cortico-striatal-thalamo-cortical integration in humans, further evidence by functional and connectivity studies is needed to substantiate these first results.

## Clinical perspectives of high-resolution thalamic imaging

The concept of functional integration of segregated thalamo-cortical loops—and therefore thalamic nuclei—could for a long time not be substantiated by human imaging findings. Therefore, clinical concepts of thalamo-cortical integration were often based on animal work, post-mortem or human lesion studies, but lacked the integration of human functional (imaging) findings. As one example, the controversial findings of stronger or weaker functional connectivity between different parts of the cingulate cortex and “the thalamus” as a whole—as described in various patient populations (Anand et al., 2005; Greicius et al., 2007; Minzenberg et al., 2009; Walter et al., 2009)—could not be traced back to distinct thalamic nuclei. Supported by animal studies, some thalamic structures are, however, targeted by deep brain stimulation in various diseases like depression, obsessive compulsive disorder (see Lujan et al., 2008 for an extensive review) and Parkinson's disease (Fasano et al., 2012).

While circumscribed anatomical localization of thalamic structures still remains a challenge in MRI-guided neurosurgery, thalamic nuclei represent anatomically well-defined structures with small inter-subject variability—as compared to that found to a much larger extent in cortical brain regions. As one example, Sartorius and colleagues recently showed an antidepressant effect of electrode placement near the lateral habenular complex for deep brain stimulation purposes (Sartorius and Henn, 2007; Sartorius and Meyer-Lindenberg, 2009; Sartorius et al., 2010).

The lateral habenular complex neighbors the thalamic structures described above, and deep brain stimulation is not restricted to one point in the brain, but rather acts in a radius within a sphere of brain tissue. Therefore, the functional diversity and connecting fibers of neighboring thalamic nuclei in humans requires a larger knowledge.

As for MD and CM such functional diversity has been introduced as described in the previous paragraphs, this sheds a different light on the explanation of effectivity of thalamic targets in deep brain stimulation. While CM is a thalamic region targeted to treat epilepsy with the help of deep brain stimulation (Cukiert et al., 2009), its stimulation has also been described as an effective treatment option against Parkinson's disease (Stefani et al., 2009) and a promising target in Tourette's syndrome, movement disorders (Krauss et al., 2002; Ackermans et al., 2011) and pain syndromes (Weigel and Krauss, 2004).

As thalamus and the basal ganglia form the major targets of neurostimulation in humans—the investigation of their integration into cortico-striato-thalamo-cortical loops seems crucial not only for the optimization of current treatment practice, but also for the development of new treatment options. With the help of high-resolution imaging at ultra-high fields, such crucial knowledge can now be gained (Duchin et al., 2012) and provide new insights in human brain function during health and disease.

Based on the finding by Eckert and colleagues, Osoba and colleagues looked at differences in MD and CM fiber tracts between patients with major depression and healthy controls (Osoba et al., 2013). As stated above, the MD was more closely linked to emotional perception, while attentional processing was more associated with CM thalamus (Metzger et al., 2010). As both emotion and attention processes have been found abnormal in MDD patients, the comparison of fiber tracts of MD and CM with their putative preferential targets seemed quite intuitive, especially as altered function and histology of the MD has been previously shown in MDD (Drevets et al., 1992; Steiner et al., 2008). Indeed, the distinction of structural disconnections of CM and MD turned out as a meaningfull level of observation. Osoba and colleagues reported significant differences for fractional anisotropy (FA) in an MDD group compared to healthy controls, however, only in tracts of interest that were defined by the previously reported preferential networks of MD and CM (Eckert et al., 2011). Reductions in FA were thus restricted to those tracts originating from CM or MD, which connected them with cortical and subcortical target structures that would also preferentially connect to these nuclei in healthy controls.

## Conclusion

At sufficiently high resolution, consistency between human and animal anatomy and function increases. Therefore, and for the fact that high resolution imaging is reliable and feasible, it should be aimed to more and more implement high resolution imaging in human basic and clinical imaging—especially as the anatomical basis for human stimulation studies, is built on models on nuclei level. Current work at spatial resolutions of 2 mm^3^ voxel size or lower provided support for a meaningful level of observation that is not accessible at lower resolutions. This applied for both basic physiological studies in healthy populations as well as for patient populations. The functional distinctions, revealed by such experiments further converged with structural findings, which, also at lower fields are able to investigate thalamic connectivity at the level of anatomically separable nuclei. Especially when higher resolution is needed for subcortical structures, group studies with an acceptable between subject alignment seem possible. When investigating cortico-striato-thalamo-cortical circuits, future research should thus incorporate the notion that such spatial resolution, so far sufficient to discern cortical subregions of larger extent, need to be adapted for the smallest functional units included in the circuit. This should enable and encourage more investigations that focus on related thalamo-cortical networks in humans and should help to disambiguate findings arising from studies with insufficient resolution for subcortical network components.

### Conflict of interest statement

The authors declare that the research was conducted in the absence of any commercial or financial relationships that could be construed as a potential conflict of interest.

## Key Concept

Cortico-striatal-thalamo-cortical circuits: The concept of segregated but integrated circuits, forming functional subsystems or cortico-striatal-thalamo-cortical “loops,” was suggested by Alexander and others, mainly based on animal studies (Alexander et al., 1986). These circuits connect functionally segregated, higher order cortical regions with the basal ganglia and form functional entities for motor and sensory processes as well as for higher cognitive functions like cognitive and emotional processes.
Ultra-high resolution imaging: High magnetic field strength results in an increased signal to noise ratio and an expansive increase of the sensitivity to detect BOLD changes (Gati et al., 1997). Superiority over lower field strengths has been established more and more over the last years (Pfeuffer et al., 2002). It enables the functional investigation of very small, anatomically well-described subcortical structures in humans.
Mediodorsal thalamic nucleus (MD): As main part of the limbic thalamus, the MD forms a thalamic relay station linking basal ganglia and cortex (Vogt and Pandya, 1987; McFarland and Haber, 2002). It was shown to play a major role in cognition, emotion, memory, and motivation in animals (Haber and McFarland, 2001) and was linked to the processing of emotionally salient stimuli in humans (Walter et al., 2008b; Metzger et al., 2010).
Centromedian/parafascicular thalamic complex (CM/PF): The CM/PF forms the major part of the intralaminar nuclei (van der Werf et al., 2002). As one major function, it has been related to attentional processing in animals (Kinomura et al., 1996) and more specific to the attentional shift to motivationally relevant stimuli in animals (Matsumoto et al., 2001; van der Werf et al., 2002) and in humans (Metzger et al., 2010).
Preferential connectivity: It describes a relative preference of fiber tract distributions of a region A with the two distinct regions B and C. If the connectivity of A with B is stronger than with C, then A is preferentially connected to B. The same not only accounts for preferential anatomical connectivity, but also preferential functional connectivity during task or restingstate conditions (e.g., Eckert et al., 2011).
